# Improved Brain–Computer Interface Signal Recognition Algorithm Based on Few-Channel Motor Imagery

**DOI:** 10.3389/fnhum.2022.880304

**Published:** 2022-05-06

**Authors:** Fan Wang, Huadong Liu, Lei Zhao, Lei Su, Jianhua Zhou, Anmin Gong, Yunfa Fu

**Affiliations:** ^1^Faculty of Information Engineering and Automation, Kunming University of Science and Technology, Kunming, China; ^2^Brain Cognition and Brain-Computer Intelligence Integration Group, Kunming University of Science and Technology, Kunming, China; ^3^Faculty of Science, Kunming University of Science and Technology, Kunming, China; ^4^School of Information Engineering, Chinese People’s Armed Police Force Engineering University, Xi’an, China

**Keywords:** MI-BCI with fewer channels, Dempster–Shafer evidence theory, time-frequency decomposition (TFD), phase space reconstruction (PSR), common spatial pattern (CSP)

## Abstract

Common spatial pattern (CSP) is an effective algorithm for extracting electroencephalogram (EEG) features of motor imagery (MI); however, CSP mainly aims at multichannel EEG signals, and its effect in extracting EEG features with fewer channels is poor—even worse than before using CSP. To solve the above problem, a new combined feature extraction method has been proposed in this study. For EEG signals from fewer channels (three channels), wavelet packet transform, fast ensemble empirical mode decomposition, and local mean decomposition were used to decompose the band-pass filtered EEG into multiple time–frequency components, and the corresponding components were selected according to the frequency characteristics of MI or the correlation coefficient between its time–frequency components and the original EEG signal. Furthermore, phase space reconstruction (PSR) was performed on the selected components after the three time-frequency decompositions, the maximum Lyapunov index was calculated, and the features were reconstructed; then, CSP projection mapping was used for the reconstructed features. The support vector machine probability output model was trained by the obtained three mappings. Probability outputs by three different support vector machines were then obtained. Finally, the classification of test samples was determined by the fusion of the Dempster–Shafer evidence theory at the decision level. The results showed that the accuracy of the proposed method was 95.71% on data set III of BCI competition II (left- and right-hand MI), which was 2.88% higher than the existing methods. On data set IIb of BCI competition IV, the average accuracy was 86.60%, which was 2.3% higher than the existing methods. This study verified the effectiveness of the proposed method and provided an approach for the research and development of the MI-BCI system based on fewer channels.

## Introduction

Brain–computer interface (BCI) is a revolutionary human–computer interaction with the ability to improve the quality of life of medical patients, disabled people, and healthy people (Wolpaw et al., 2000; Cheng et al., 2002; Blankertz et al., 2010). Among various types of BCI, motor imagery (MI) is an important BCI paradigm with great potential to be applied in rehabilitation training for motor dysfunction (Attallah et al., 2020).

In MI-BCI, effectively decoding MI intent based on electroencephalogram (EEG) signals is an important problem (Chen et al., 2018; Wang et al., 2018; Jiao et al., 2019; Zhang et al., 2019). Common spatial pattern (CSP) is often used to extract features in most MI-BCI studies; this algorithm is recognized as an effective method to extract features of MI-EEG (Ang et al., 2008; Wang et al., 2017; Jin et al., 2021; Miao et al., 2021). However, CSP is mainly applicable to multichannel EEG signals and is not effective in extracting EEG features with fewer channels for identification. In addition, the application of multichannel MI-BCI is limited due to a large number of channels. In comparison, MI-BCI with fewer channels is more practical. For MI-BCI with fewer channels, how to effectively apply CSP to this kind of BCI is a problem worth exploring.

Although CSP has many improved methods in multichannel MI-BCI and achieved good results, these improved algorithms cannot be directly used in few-channel MI-BCI. Moreover, in the case of MI-BCI with fewer channels, due to the lack of sufficient spatial information, the classification accuracy of features extracted by CSP is worse than other simple spatial filtering methods. To solve this problem, Huang et al. (Huang, 2009) regarded multiple frequency bands of each channel as new channels and used CSP to extract features. As such, this method can effectively utilize frequency domain information in the case of fewer channels. However, it cannot completely replace spatial information. Meng et al. (2013) used multiple coordinate delays of each channel time series to optimize a spatial filter and a higher order multiparameter FIR filter at the same time, thereby enhancing the distinguishability of MI-EEG signals with fewer channels. Nevertheless, the results of this method are closely related to the selection of the number of delay factors, and the number of delay factors is generally larger, which will seriously affect the computational complexity of this method. Yang et al. (2015) used phase space reconstruction (PSR) to decompose EEG with fewer channels into multichannel signals, but the effect of PSR mainly depends on the delay parameters. However, the selection of embedded dimension parameters has less effect on its performance, as PSR is directly used to expand the channel, and the reconstructed signal still has the problem of insufficient spatial information. Guan and Duan (2019) put forward a spectral feature and transformation method based on multivariate empirical mode decomposition (MEMD), which has some advantages while suffering from some problems such as high computational complexity and insufficient spatial information after decomposition and reconstruction. Xu et al. (2019) used wavelet transform and CNN to decode MI-EEG signals. Although this method can improve the classification accuracy of MI-BCI to a certain extent, it requires a large number of training data sets and is not suitable for the study of a few-channel MI-BCI. Wang and He (2004) used means of frequency decomposition and weighting synthesis strategy for recognizing imagined right- and left-hand movements. Although this method has a certain effect on MI-BCI signal decoding, it is only suitable for multichannel MI-EEG decoding and cannot effectively solve the problem of insufficient spatial information of few-channel MI-EEG signals.

In view of the above problems of CSP applied to MI-BCI with fewer channels, this study proposed a combined feature extraction method called TFD-PSR-CSP. This method utilizes different time–frequency decomposition (TFD) methods (involving wavelet packet transform [WPT], fast ensemble empirical mode decomposition [FEEMD, and local mean decomposition [LMD]) combined with PSR, which expands the EEG signals from fewer channels into multichannel EEG. Finally, features were extracted by CSP. Afterward, considering the nonlinear and nonstationary characteristics of EEG signals, in this study, three different support vector machine (SVM) probability outputs were obtained by introducing a sigmoid function for probability mapping of SVM outputs. Finally, the Dempster–Shafer (D–S) evidence theory is used for decision-making fusion to determine the category of test samples. This study is organized as “Materials and methods,” “Results,” “Discussion,” and “Conclusion” sections.

## Materials and Methods

### EEG Data Set and Preprocessing

#### EEG Data Set

To evaluate the performance of TFD-PSR-CSP and D–S evidential theory in the identification of MI intention with fewer channels, data set III of BCI competition II (denoted as data set 1) and data set IIB of BCI competition IV (denoted as data set 2) are adopted, which are briefly described as follows:

Data set 1: A female subject, aged 25 years, in good health performed left- and right-hand MI; and a single trial lasted for 9 s, totaling 280 trials, all of which were completed on the same day and were divided into seven groups. Three bipolar EEG channels were recorded before and after C3, CZ, and C4. The EEG sampling frequency was 128 Hz, and band-pass filtering was performed at 0.5–30 Hz. Finally, the data set is distributed equally into training and testing sets, each consisting of 140 trials.
For additional details, see http://www.bbci.de/competition/ii/.

Data set 2: In total, nine healthy subjects, all of whom had normal eyesight or eyesight corrected to normal, and all of them were right-handed. All subjects participated in five groups (five sessions) of the left- and right-hand MI experiments, among which the first two sessions had no feedback and the last three sessions had feedback. Each subject conducted 160 trials with feedback (80 trials for the right hand, and 80 for the left hand). Three bipolar leads (C3, CZ, and C4) were recorded with a sampling frequency of 250 Hz, 0.5–100 Hz band-pass filtering, and 50 Hz notch processing. According to some studies (Zhang et al., 2019) and (Bustios and Rosa, 2017), only the third session data were used for evaluation. Each subject performed 160 trials, i.e., 80 trials per class. For additional details, see http://www.bbci.de/competition /iv/.

#### Pretreatment

In this study, for each trial of data set 1, the data segment of 0.5–3 s after the prompt was extracted; for each trial of data set 2, the data segment of 0–4 s after the prompt was extracted, and the prompt time was 0 s. Studies have shown that the frequency range of EEG-based MI-BCI signals is generally 8–30 Hz; so in this study, the original EEG data were filtered at 8–30 Hz by the 5th-order Butterworth band-pass filter.

### Method

#### General Idea and Fusion Algorithm Flow

**FIGURE 1:**
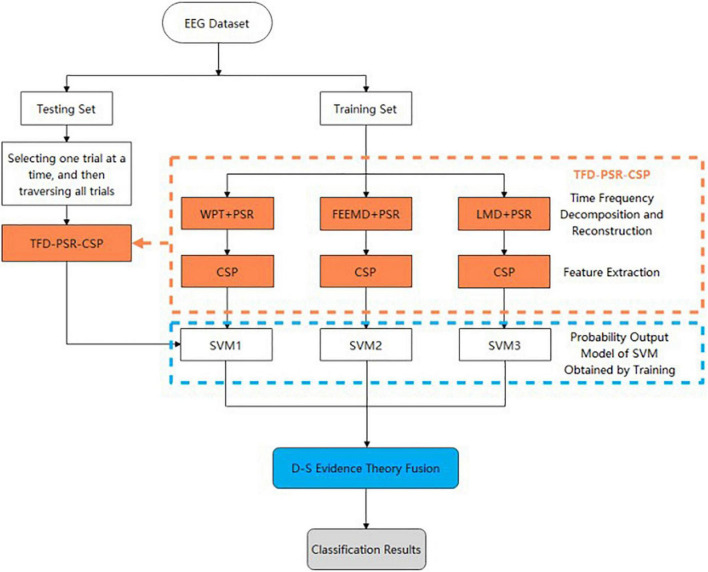
Flowchart of fusion algorithm proposed in this study.

The overall research idea and fusion algorithm flow of the proposed method are shown in Figure 1. First, the EEG data set was divided into a training set and a testing set, and TFD-PSR-CSP was used to extract features from the training data set. Specifically, WPT + PSR, FEEMD + PSR, and LMD + PSR were used for time–frequency decomposition and reconstruction, and features were extracted by CSP to construct feature vectors. Then, the SVM classifier was trained. A sigmoid function was introduced to map the classification results to probability, and three SVM probability output models were obtained through training.

After establishing the probability output model of SVM on the training set, TFD-PSR-CSP extracted features from any trial on the test set. Then, the probability output of each category of the test sample was obtained through the trained SVM probability output model. Finally, the classification result was obtained through D–S evidence theory fusion.

#### TFD-PSR-CSP Method and Its Specific Design

TFD-PSR-CSP first carried out TFD on the preprocessed EEG data to obtain effective time–frequency information, then carried out PSR to expand the EEG signal with few channels into multichannel EEG, and finally extracted features with good separability by CSP. The method and specific design are described below.

(1) Time–frequency decomposition (TFD)

In this experiment, three different TFD methods were used: WPT, FEEMD, and LMD.

(1) Wavelet packet transform (WPT)

WPT is an adaptive time–frequency localization analysis method whose time window and frequency window can be changed (Ting et al., 2008). The sampling frequencies of data set 1 and data set 2 in this study were 128 and 250 Hz, respectively. According to the Nyquist sampling theorem, the best decomposition layers of WPT were layers 3 and 4. For an arbitrary trial on data set 1 and data set 2, WPT was performed on the EEG signals of C3, C4, and CZ channels, and the db4 wavelet was used after comparing the results of each wavelet base.

**FIGURE 2:**
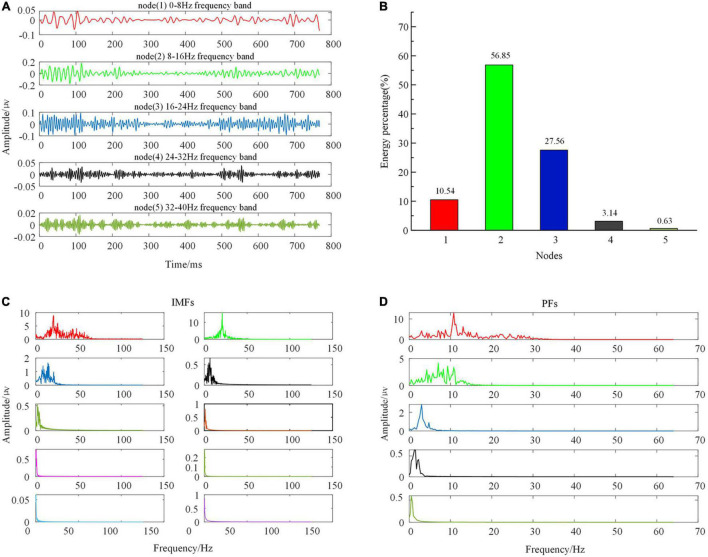
Time domain, frequency spectrum, and energy ratio diagram of each component of EEG signal after TFD. **(A)** EEG waveform of the first 5 nodes of layer 3 after WPT; **(B)** EEG energy ratio diagrams of the first 5 nodes of layer 3 after WPT; **(C)** Spectrum diagram of each IMF component after FEEMD; **(D)** Spectrum diagram of each PF component after LMD.

Each node in the 3rd or 4th layer was reconstructed, the energy ratio in each frequency range was calculated, and the related node signal was selected as the object of subsequent processing. In this way, the original 3-channel (C3, CZ, and C4) signals were expanded into 12-channel EEG data after WPT. Figures 2A,B illustrates the EEG waveform and the energy ratio of the first five nodes in layer 3 after WPT of the C3 channel of the EEG signal in data set 1.

(2) Fast ensemble empirical mode decomposition (FEEMD)

Fast ensemble empirical mode decomposition is suitable for processing nonlinear and nonstationary signals, which can effectively solve the mode aliasing problem in empirical mode decomposition (EMD) and further reduce the computational complexity (Wang et al., 2014; Chen and You, 2017; Dai et al., 2021). In this experiment, FEEMD was used to decompose the preprocessed EEG data in time and frequency, which mainly involved setting two parameters, namely, the amplitude coefficient of the white noise sequence and the number of times EMD was executed.

For convenience, taking the EEG signal of the left-hand MI C3 channel of data set 1 as an example, the amplitude coefficient of the white noise sequence was determined to be 0.2 by the grid search method, and EMD was executed 40 times. Each channel to be decomposed was divided into 10 intrinsic mode function (IMF) components. It can be seen from the spectrogram of each component in Figure 2C that the frequency ranges of IMF1, IMF2, IMF3, and IMF4 were 8–30, 8–16, 0–12, and 0–6 Hz, respectively, and the amplitude of each component decreased with the increase of decomposition number. Comparing the frequency bands of these four components and considering the frequency range of MI-EEG, the first three components (IMF1–IMF3) were selected to form a new EEG signal as the object of subsequent processing. In this way, the original 3-channel (C3, CZ, and C4) signals were expanded into 9-channel EEG data after passing through FEEMD.

(3) Local mean decomposition (LMD)

Local mean decomposition is an adaptive time–frequency analysis method for nonstationary signals proposed by Smith (Smith, 2005; Liu et al., 2018). The original signal *x*(*t*) can be expressed as the sum of the product function *PF_i_*(*t*) and the residual *u_n_*(*t*), as follows:


(1) $$x\,\left(t\right)=\sum_{i=1}^{n}{P\,F}_{i}\left(t\right)+u_{n}\left(t\right).$$

For convenience, taking the EEG signal of channel C3 of data set 1 as an example, first, the preprocessed EEG signal is decomposed into several PF components by LMD, as shown in Figure 2D. In Figure 2D, the frequency ranges of PF1, PF2, and PF3 are 0–30, 0–16, and 0–7 Hz, respectively, and the frequency ranges of PF4 and PF5 are significantly lower than 8 Hz. Considering the frequency range of MI-EEG and calculating the correlation coefficient between EEG data before LMD and PF components after LMD, the correlation coefficients with PF1, PF2, and PF3 are 0.9194, 0.2420, and 0.0015, respectively. Therefore, PF1 and PF2 are selected to form a new EEG signal as the subsequent processing object. In this way, the original 3-channel (C3, CZ, and C4) signals are expanded into 6-channel EEG data after LMD.

The numbers of expanded channels of the original EEG data after WPT, FEEMD, and LMD are 12, 9, and 6, respectively. The following PSR is used to further expand the spatial information.

(2) Phase space reconstruction (PSR)

Electroencephalogram signals have chaotic characteristics. PSR is an effective method for analyzing chaotic time series, and studies have shown that it is suitable for extracting MI-EEG features (Meng et al., 2013). Takens (1981) put forward the embedding theorem and used the delayed coordinate method to analyze chaotic time series *x* = {_*x(i)|i*_ = 1,2,….*N*} and carry out reconstruction. The resulting reconstructed signal *y*(*i*) is


(2) y⁢(i)={x⁢(i),…⁢…⁢x⁢(i+(d-1)⁢τ)}⁢1≤i≤n-(d-1)⁢τ,

where τ is time delay, and *d* is embedded dimension. In this study, the C-C method proposed by Kim et al. (1999) is used to select the values of τ and *d*.

To clearly explain how WPT + PSR, FEEMD + PSR, and LMD + PSR are formed, the original 3-channel MI-EEG data were reconstructed by WPT, FEEMD, and LMD, and the number of channels was expanded to 12, 9, and 6 channels, respectively. Although these three TFD methods expanded the spatial information of EEG with few channels, the expansion is limited. To further expand the spatial information of EEG, we use the C-C method proposed by Kim et al. based on the idea of the embedded window method to calculate the time delay τ and embedded dimension *d* of the above three kinds of TFD EEG data, and then we use the searching method to select the best parameter values. Then, based on the optimal parameter values and the actual computational complexity, the embedding dimensions of WPT, FEEMD, and LMD processed data were selected as 2, 2, and 3, respectively, by PSR. In this way, the EEG data after three different TFDs were further processed by PSR, and the number of channels was extended to 24, 18, and 18 channels, respectively.

(3) Common spatial pattern (CSP) algorithm

After the original 3-channel EEG signal was expanded into a multichannel signal by TFD-PSR, CSP was proposed to extract features.

The main idea of the CSP algorithm is to diagonalize two kinds of covariance matrices at the same time, i.e., maximize one kind of variance and minimize the other kind of variance, to find a set of optimal spatial filters and obtain the feature vector with the best separability by projection. Before the feature extraction algorithm is applied, band-pass filtering and centralized processing are generally required for data, so the spatial covariance matrix of these two types of data is expressed as:


(3) $$C_{1}=\frac{1}{n_{1}}\,\sum_{i=1}^{n_{1}}X_{i\,1}X_{i\,1}^{T}C_{2}=\frac{1}{n_{2}}\,\sum_{i=1}^{n_{2}}X_{i\,2}X_{i\,2}^{T},$$

where *X*_*i*,1_,*X*_*i*,2_ ∈ R^N*T^ represent the two types of EEG data obtained from the *i*-th experiment, *N* represents the number of channels, *T* represents the number of sampling points, and *n*_1_ and *n*_2_ are used to represent the number of experiments of these two categories.

The mathematical principle of the CSP algorithm is to maximize the following objective function (Lotte and Guan, 2011):


(4) $$J\,\left(w\right)=\left(\frac{w^{T}c_{1}w}{w^{T}c_{2}w}\right)\,s.t.{||w||}_{2}=1$$

where *w* is the spatial filter.

The optimization problem of the Rayleigh quotient can be transformed into the generalized eigenvalue problem. By solving the generalized eigenvalue problem, the optimal spatial filter can be obtained.


(5) $$c_{1}w=\lambda c_{2}w,$$

where λ is a generalized eigenvalue, and *w* is a generalized eigenvector. The eigenvalue λ is used to calculate the variance ratio between the two classes. The spatial filter *w_csp_* is composed of *n* eigenvectors corresponding to maximum and minimum eigenvalues.

Finally, the original signal *X_i_* is passed through the spatial filter *w_csp_* to obtain the projection signals *Z_i_* :


(6) $$Z_{i}=w_{c\,s\,p}^{T}X_{i}.$$

#### D–S Evidence Theory

After features are extracted by TFD-PSR-CSP, the sigmoid function is used to map SVM outputs to obtain three different SVM probability outputs, and D–S evidence theory is used to determine the classification of the final test samples by decision-level fusion.

Dempster–Shafer evidence theory is a decision-making method for uncertain reasoning proposed and improved by Dempster and Shafer (Dempster, 1967; Shafer, 1976; Jin et al., 2021). It combines multiple pieces of evidence by rules to make decisions under a known identification framework to obtain higher correct recognition and reliability (Mathon et al., 2010).

To use the D–S evidence theory for decision-level fusion in an MI-EEG signal classification problem, this experiment introduces the sigmoid function to map the probability (Platt, 1999) of an EEG sample category output by SVM belonging to the left-handed MI category {L} or the right-handed MI category {*R*}. The recognition framework is defined as follows:


(7) Θ={{L},{R}}.

The power series 2^Θ^ of the identification framework is as follows:


(8) $$2^{\Theta }=\left\{\emptyset ,\left\{L\right\},\left\{R\right\},\Theta \right\},$$

where ∅ is an empty set. According to the trained SVM probability output model, the corresponding probability *P*{*L*} or *P*{*R*} of the test sample category {L} or {*R*} is obtained, and then the basic probability assignment (BPA) value of each focal element under the recognition framework is calculated by formulas (9)–(11).


(9) $$S\,\left(\left\{L\right\}\right)=P\,\left(\left\{L\right\}\right)\,\left(1-\frac{N_{s\,v}}{l-1}\right).$$

(10) $$S\,\left(\left\{R\right\}\right)=P\,\left(\left\{R\right\}\right)\,\left(1-\frac{N_{s\,v}}{l-1}\right).$$

(11) $$S\,\left(\Theta \right)=\frac{N_{s\,v}}{l-1}.$$

where *S*({*L*}) and *S*({*R*}) represent the BPA values of categories {L} and {R}, respectively, *S*(Θ) represents the BPA values of the identification framework, *N_sv_* represents the number of support vectors in the SVM probabilistic output model, and *l* represents the total number of samples.

After obtaining the BPA value of each focal element under the identification framework, the BPA values of each focal element are fused by D-S evidence theory combination rules (12), (13). Assuming that ∀*A*⊂Θ exists, the combination rules of *n* mass functions in the recognition framework Θ are as follows:


(12) $$\begin{array}{c} \left(m_{1}⊕m_{2}\ldots \,m_{n}\right)=\frac{1}{K}\,\sum_{A_{1}\cap A_{2}\cap A_{n}\neq \emptyset }m_{1}\left(A_{1}\right)*m_{2}\left(A_{2}\right)*m_{n}\left(A_{n}\right) \end{array},$$

(13) $$\begin{array}{c} K=\sum_{A_{1}\cap A_{2}\ldots \cap A_{n}\neq \emptyset }m_{1}\left(A_{1}\right)*m_{2}\left(A_{2}\right)\ldots \,m_{n}\left(A_{n}\right) \end{array}$$

$$=1-\frac{1}{K}\,\sum_{A_{1}\cap A_{2}\cap A_{n}=\emptyset }m_{1}\left(A_{1}\right)*m_{2}\left(A_{2}\right)*m_{n}\left(A_{n}\right).$$

In these formulas, *K*is the normalization factor, and $1-\frac{1}{K}$ indicates the conflict degree of evidence. The mass function indicates the trust degree of each category after classification, and the trust level of category *A_i_* is *m*(*A_i_*). When expressed by probability, *m*(*A_i_*) represents the probability of class *A* in the *i-*th probability output model, and *A_i_* represents class *A* in the *i-*th probability output model.

After obtaining the fused BPA values *S_f_*({*L*}), *S_f_*({*R*}), and *S_f_*({Θ}), the classification of the final test samples is determined according to the following decision rules:

Decision rules: If a test sample belongs to class Ω, Ω ∈ {{*L*},{*R*}}, therefore should satisfy the following conditions:


$$\left(1\right)\,S_{f}\left(\Omega \right)=\max\left(S_{f}\left(\left\{L\right\}\right),S_{f}\left(\left\{R\right\}\right),S_{f}\left(\left\{\Theta \right\}\right)\right);$$

$$\left(2\right)\,S_{f}\left(\Omega \right)-S_{f}\left(\Lambda \right)>\varepsilon_{1},\varepsilon_{1}\in \left\{\left\{L\right\},\left\{R\right\}\right\}\,a\,n\,d\,\varepsilon_{1}\neq \Omega ;$$

$$\left(3\right)\,S_{f}\left(\Theta \right)<\varepsilon_{2}.$$

In this experiment, ε_1_ = 0, ε_2_ = 0.1.

## Results

### Classification Accuracy of TFD-PSR-CSP Combined With D–S Evidence Theory

The last column in Table 1 shows the classification accuracy of the proposed method on data set 1 and data set 2, which are 95.71 and 86.60 ± 11.14%, respectively.

**TABLE 1:** 5 × 5 cross-validation classification accuracy of proposed methods and different methods using BCI competition II data set III and competition IV data set IIb.

Data set	Participant	Method						
		CSP	FEEMD + CSP	LMD + CSP	WPT + CSP	PSR + CSP	FDM	TFD-PSR-CSP + D-S evidence theory
Data set 1	a	84.29	91.43	87.14	90	88.57	92.86	95.71
Data set 2	b	77.5	82.5	81.25	82.25	83.75	82.5	86.25
	c	55.83	61.67	64.17	63.33	59.17	56.67	68.51
	d	56.25	66.25	63.75	62.5	61.25	60	68.75
	e	90	97.5	95	96.25	91.25	90	100
	f	86.25	91.25	92.5	92.5	90	86.25	96.25
	g	81.25	85	86.25	87.5	87.5	88.75	91.25
	h	76.75	81.25	82.5	80	80	75	83.75
	i	83.75	87.5	86.25	87.5	86.25	81.25	92.5
	j	80	88.75	87.5	88.75	82.5	86.25	93.75
	Mean ± SD	76.40 ± 12.26	82.41 ± 11.57	82.13 ± 11.16	82.29 ± 12.0	80.21 ± 11.90	78.52 ± 12.31	86.60 ± 11.14
	*P* value		*P* < 0.001	*P* < 0.001	*P* < 0.001	*P* < 0.001	0.0547	*P* < 0.001

**FIGURE 3:**
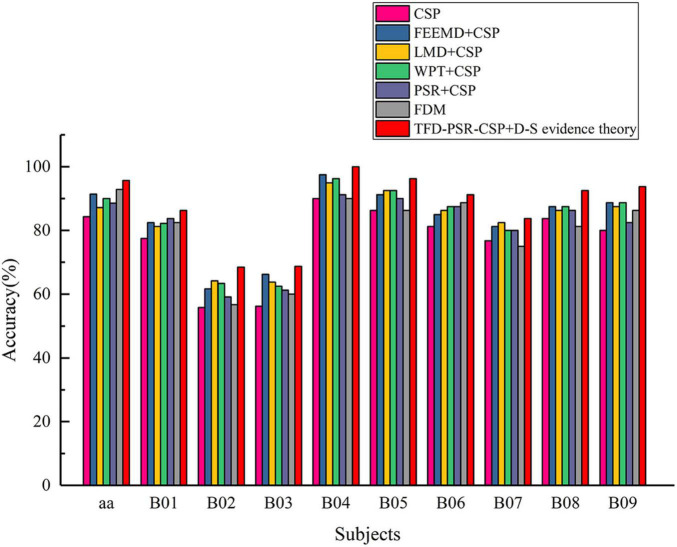
Classification accuracy of all participants in data set 1 and data set 2.

Figure 3 shows the classification accuracy of the proposed method, traditional CSP, and different time–frequency decomposition and reconstruction methods. The results show that TFD and PSR achieve better classification accuracy than traditional CSP methods by effectively combining with CSP. At the same time, the proposed method in this study achieves the highest classification accuracy in all subjects.

### Kappa Value of TFD-PSR-CSP Combined With D–S Evidence Theory

In the last column of Table 2, the kappa values of the proposed method in data set 1 and data set 2 are given, which are 0.91 and 0.74 ± 0.229, respectively.

**TABLE 2:** 5 × 5 cross-validation kappa value of proposed methods and different methods using BCI competition II data set III and competition IV data set IIb.

Data set	Participant	Method						
		CSP	FEEMD + CSP	LMD + CSP	WPT + CSP	PSR + CSP	FDM	TFD-PSR-CSP + D-S evidence theory
Data set 1	a	0.69	0.83	0.75	0.80	0.77	0.86	0.91
Data set 2	b	0.55	0.65	0.63	0.65	0.68	0.65	0.73
	c	0.12	0.23	0.28	0.27	0.18	0.13	0.37
	d	0.13	0.33	0.28	0.25	0.23	0.20	0.38
	e	0.8	0.95	0.9	0.95	0.83	0.8	1
	f	0.73	0.83	0.85	0.85	0.8	0.73	0.93
	g	0.63	0.7	0.73	0.75	0.75	0.78	0.83
	h	0.53	0.63	0.65	0.6	0.6	0.5	0.65
	i	0.68	0.75	0.73	0.75	0.73	0.63	0.85
	j	0.6	0.77	0.75	0.77	0.65	0.73	0.88
	Mean ± SD	0.53 ± 0.245	0.65 ± 0.231	0.64 ± 0.223	0.65 ± 0.243	0.61 ± 0.238	0.57 ± 0.248	0.74 ± 0.229
	*P* value		*P* < 0.001	*P* < 0.001	*P* < 0.001	*P* < 0.001	0.0557	*P* < 0.001

**FIGURE 4:**
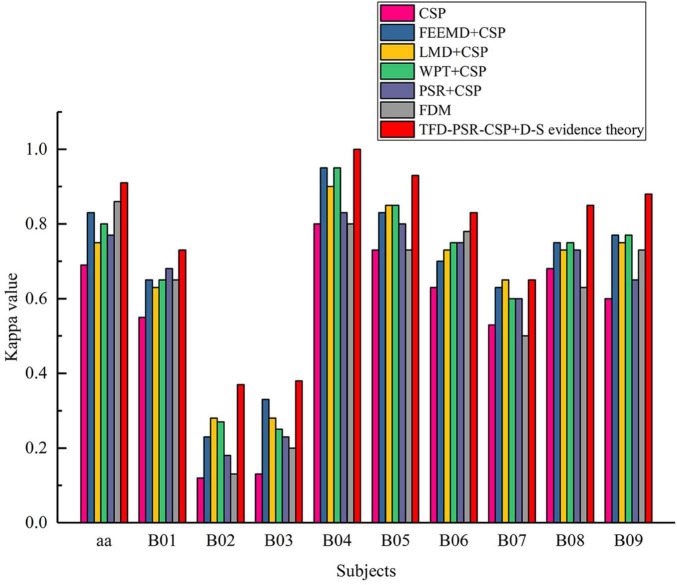
Kappa values of all participants in data set 1 and data set 2.

Figure 4 shows the kappa value of the proposed method, traditional CSP, and different time–frequency decomposition and reconstruction methods. The results show that except FDM method, other methods obtain higher kappa values than traditional CSP. At the same time, the proposed method in this study achieves the highest kappa value in all subjects.

## Discussion

To make better use of the D–S evidence theory for decision-level fusion to get the final test sample category, this study employs three different TFD methods with PSR-CSP + SVM training to get three different probability output models. The following compares the proposed method with traditional CSP and different time–frequency decomposition reconstruction methods and also with other existing methods.

### Comparison of TFD-PSR-CSP and D-S Evidence Theory With Traditional CSP and Different Time–Frequency Decomposition Reconstruction Methods

#### Classification Accuracy

It can be seen from Table 1 that the classification accuracy of the proposed method was improved from 84.29 to 95.71% compared with the traditional CSP on data set 1. In addition, before extracting features from EEG data by CSP, using PSR, the degree was increased from 84.29 to 88.57%, which indicated the effectiveness of PSR in the intention identification of MI with fewer channels. TFD not only expanded the number of channels of original EEG data but also achieved better classification accuracy when combined with CSP than traditional CSP; this also showed the effectiveness of TFD in the intention identification of MI with fewer channels.

It can also be seen from Table 1 that on data set 2, the average classification accuracy of the method proposed in this study changed from 78.52 ± 12.31 to 86.60 ± 11.14% compared with FDM (direct fusion of features extracted from three different TFDs combined with PSR-CSP). In addition, the classification accuracy of traditional CSP and other methods was tested by the paired *t*-test, and the *P-*values of other methods were far less than 0.005, except for FDM (*P*-value is 0.0547). The above showed that the fusion of D–S evidence theory after extracting features by different TFDs combined with PSR-CSP was better than FDM.

#### Kappa Value

It can be seen from Table 2 that the kappa value or mean value of the proposed method was increased by 0.22 and 0.21, respectively, compared with the traditional CSP on data sets 1 and 2. In addition, before extracting EEG features by CSP, TFD, and PSR were used to expand the number of original EEG data channels to increase the amount of spatial information, and the kappa value or mean value was improved. This showed the effectiveness of TFD and PSR in the intention identification of MI with fewer channels. Compared with FDM, the kappa values of data sets 1 and 2 increased from 0.86 to 0.91 and from 0.57 to 0.74, respectively, which indicated that the D–S evidence theory fusion was superior to FDM after different TFD and PSR combined with CSP to extract features.

#### Feature Distribution

**FIGURE 5:**
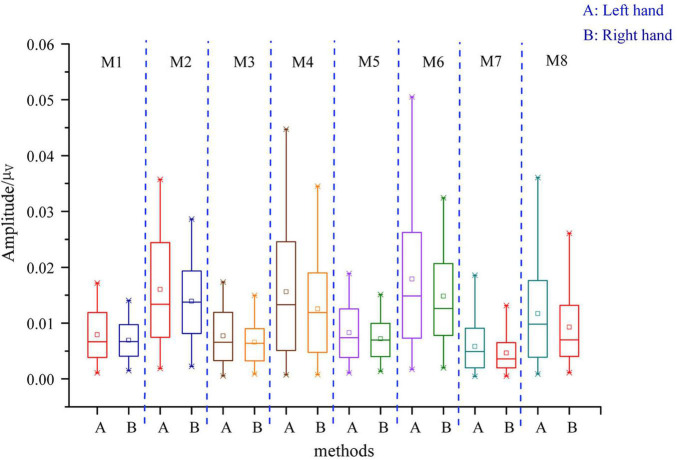
Characteristic box diagram of traditional CSP and of three different time–frequency decomposition and reconstruction methods. M1 represents the characteristic box diagram of CSP; M2 represents the characteristic box diagram of PSR + CSP; M3 represents the characteristic box diagram of WPT + CSP; M4 represents the characteristic box diagram of WPT + PSR + CSP; M5 represents the characteristic box diagram of LMD + CSP; M6 represents the characteristic box diagram of LMD + PSR + CSP; M7 represents the characteristic box diagram of FEEMD + CSP; M8 represents the characteristic of box diagram FEEMD + PSR + CSP.

To compare the distribution differences of features extracted by different methods, Figure 5 presents the distribution of features extracted by these methods on data set 1 (for convenience, only the two preferred features in each method are displayed). It can be seen from Figure 5 that the feature distribution obtained by combining three different TFD methods and PSR with CSP in this study was more separable than that obtained by a single CSP.

To intuitively compare the distribution of features proposed by different methods, Table 3 shows the upper quartile difference and median difference of each characteristic box diagram; the difference between the upper quartile difference and the median difference indicates the separability of left- and right-hand feature distribution. The separability difference of WPT-PSR-CSP (0.00559 to –1.41e-03) was larger than the separability difference of WPT-CSP (0.00293 to –1.82e-04), which indicated that PSR can improve the separability of the left- and right-hand MI features in the intention identification of MI with fewer channels. In addition, the Fisher score (FS) was calculated for the features extracted by each method. The FS value of the proposed method was much larger than that of CSP, which also indicated that the features extracted from the original EEG by three different TFD methods and PSR combined with CSP had better separability.

**TABLE 3:** Comparison of feature distribution between CSP method and three different time–frequency decomposition reconstruction methods.

Method	Comparison mode		
	Upper quartile difference	Median difference	Fisher score
CSP	0.00216	2.67e-05	0.0086
PSR + CSP	0.00510	3.66e-04	0.0089
WPT + CSP	0.00293	−1.82e-04	0.0118
WPT + PSR + CSP	0.00559	−1.41e-03	0.0141
LMD + CSP	0.00260	−4.12e-04	0.0121
LMD + PSR + CSP	0.00557	−2.24e-03	0.0131
FEEMD + CSP	0.00257	−1.31e-03	0.0107
FEEMD + PSR + CSP	0.00443	−2.81e-03	0.0132

#### Performance Analysis of D–S Evidence Theory in Decision Fusion Layer

To analyze the performance of the D–S evidence theory in the decision fusion layer, Table 4 shows the BPA of the single-feature domain and multifeature domain fusion of two test samples. The largest BPA in the single feature domain of test sample 1 in Table 4 is *S_2_{L*} (0.5985). After the fusion of D–S evidence theory, *S*{*L*} is increased to 0.8269, and the uncertainty *S*{Θ} is decreased from the minimum of 0.1188–0.0282. Therefore, the D–S evidence theory can be used to improve BPA and reduce the uncertainty of categories.

**TABLE 4:** BPA values of test samples under the framework Θ. *S_i_* and S_i_* represent the BPA of test samples 1 and 2, respectively. s*_f_* and s_f_* represent the BPA of test samples 1 and 2, respectively, according to D–S decision fusion rules.

BPA	{*L*}	{*R*}	Θ	Classification results
*s*_1_	0.4999	0.0501	0.4500	\
*s*_2_	0.5985	0.2827	0.1188	\
*s*_3_	0.4578	0.2109	0.3313	\
*s_f_*	0.8269	0.1450	0.0282	{*L*}
*s*_1_*	0.4617	0.0883	0.045	\
*s*_2_*	0.3918	0.4894	0.1188	\
*s*_3_*	0.2927	0.3761	0.3313	\
s_f_*	0.5408	0.4241	0.0351	{*L*}

For test sample 2 in Table 4, the BPA (0.4617) of $S_{1}^{*}\left\{L\right\}$ is larger than the BPA (0.0883) of $S_{1}^{*}\left\{R\right\}$, and the BPA values of $S_{2}^{*}\left\{R\right\}andS_{3}^{*}\left\{R\right\}$ are larger than those of $S_{2}^{*}\left\{L\right\}$ and $S_{3}^{*}\left\{L\right\}$, respectively. After D–S evidence theory fusion, the BPA of $S_{f}^{*}\left\{L\right\}$ (0.5408) is higher than $S_{f}^{*}\left\{R\right\}$ (0.4241), while *S*{Θ} decreases from 0.1188 to 0.0351. The final test sample category is judged as {*L*}, which is in line with the true category of the sample. If the test sample category is judged as {*R*} according to the traditional voting principle, which is inconsistent with the true category, it shows that the fusion of D–S evidence theory can not only reduce the uncertainty of the test sample but also improve the classification accuracy.

### Comparison of TFD-PSR-CSP and D–S Evidence Theory With Existing Methods

To compare the performance of the proposed method with the existing methods (both for data set 1 and data set 2), the classification accuracy of data set 1 is compared, and the kappa value and classification accuracy of data set 2 are compared. The best accuracies and kappa values are highlighted in bold in Tables 5–7.

**TABLE 5:** Comparison of classification accuracy between the methods proposed in this study and the existing methods in data set 1.

References	Method		Classification accuracy (percentage)
	Feature extraction	Classification	
Song et al., 2016	DWT + MS-SE-FF	ELM	89.29
Kee et al., 2017	Renyi	BLDA	81.23
Zhou et al., 2018	HT + DWT	LSTM	91.43
Rodríguez-Bermúdez et al., 2013	PSR + Hjorth + AR + CWT	LOO + FLD	82.14
Ge and Hu, 2018)	TDS + AR + DWT	D-S + SVM	92.83
Wang et al., 2019	DWT + CSP	ELM	90
Guan and Duan, 2019	MEMD	LDA	88.35
Xu et al., 2019	WT	CNN	89.56
Wang et al., 2018	SC + WPT	BPNN	91
This study	TFD-PSR-CSP	D-S evidence theory + SVM	**95.71**

**TABLE 6:** Comparison of kappa values on data set 2 between the methods proposed in this study and the methods adopted by the top four in the BCI competition.

Subject	Method				
	This study	Chin	Gan	Coyle	Lodder
1	**0.73**	0.40	0.42	0.19	0.23
2	**0.37**	0.21	0.21	0.12	0.31
3	**0.38**	0.22	0.14	0.12	0.07
4	**1.00**	0.95	0.94	0.77	0.91
5	**0.93**	0.86	0.71	0.57	0.24
6	**0.83**	0.61	0.62	0.49	0.42
7	**0.65**	0.56	0.61	0.38	0.41
8	**0.85**	**0.85**	0.84	**0.85**	0.74
9	**0.88**	0.74	0.78	0.61	0.53
Mean	**0.74**	0.60	0.58	0.46	0.43

**TABLE 7:** Comparison of classification accuracy between the method proposed in this study and existing methods on data set 2.

Subject	Method								
	This study	Zhao et al., 2021	Dose et al., 2018	Wang et al., 2020	Ang et al., 2008	Zhang et al., 2019	Wang et al., 2017	Bustios and Rosa, 2017	Park and Chung, 2019
1	**88.75**	81.37	75.31	71.93	77.5	84	78.13	73.13	82.5
2	68.51	62.86	57.5	64.08	55.94	62.6	**69.03**	67.14	60
3	**68.75**	63.63	56.56	61.3	53.75	56.3	58.19	63.13	62.78
4	**100**	95.94	96.88	98.05	97.07	99.4	99.38	97.81	99.37
5	**96.25**	93.56	92.19	91.94	90.44	94.8	86.88	92.81	87.5
6	**91.25**	88.19	83.44	79.28	78.06	83.8	79.37	82.81	81.88
7	83.75	85	84.06	85.78	83.44	**94.1**	90.63	81.56	90.63
8	92.5	95.25	92.18	**93.48**	88.75	93.3	88.12	93.13	91.25
9	**93.75**	90	86.25	85.31	83.88	90.1	81.87	87.81	87.81
Average acc	**86.6**	83.98	80.56	81.24	78.75	84.3	81.29	82.15	83.15

#### Comparison of Results of Data Set 1

Table 5 compares the classification accuracy of the method proposed in this study with the existing methods on data set 1. The features adopted by these methods are different from the classification algorithm, and the results are different. It can be seen from Table 5 that the proposed method has achieved better classification accuracy, reaching 95.71%, which is 2.88% higher than the highest classification accuracy obtained by the existing method (the method proposed by Ge et al.).

#### Comparison of Results of Data Set 2

(1) Kappa value comparison

Table 6 compares kappa values of the methods proposed in this study and the methods adopted by the top four in the BCI competition on data set 2. It can be seen from Table 6 that the average kappa value of the method proposed in this study is higher than that of other methods in the table, and all subjects have achieved better performance.

(2) Comparison of classification accuracy

Table 7 compares the classification accuracy of the proposed method with the existing methods on data set 2. It can be seen from Table 7 that the proposed method has achieved better average classification accuracy, reaching 86.60%, which is 2.3% higher than the highest average classification accuracy obtained by the existing methods (the method proposed by Yu et al.), but the classification accuracy on subjects 7 and 8 is slightly lower than that of some existing methods.

Compared with multichannel MI-BCI, MI-BCI with fewer channels may be more easily accepted by users. To solve the inapplicability of CSP in MI-BCI with fewer channels, this study proposed that TFD-PSR-CSP expands the spatial information of MI-EEG with fewer channels and enriched the information in the time domain and frequency domain. In addition, the sigmoid function was used for probability mapping of SVM outputs to obtain three different SVM probability outputs; D–S evidence theory was used for decision-level fusion, which effectively improved the performance of few-channel MI-BCI.

However, the methods proposed in this study also have some limitations. One of the limitations is that compared with traditional CSP, the computational complexity of the proposed method is higher. Therefore, in our future work, we will work toward reducing the computational complexity of the proposed method. Another limitation is that the BCI competition data set was used in this study, which can only be used for offline evaluation of the proposed method. Hence, an online evaluation of the proposed method will also be a part of our future work.

## Conclusion

In view of the lack of spatial information in EEG data with fewer channels, CSP cannot extract features effectively. In this study, a time–frequency-space multidomain fusion method combining TFD-PSR-CSP feature extraction with D–S evidence theory was proposed. Compared with existing methods, it had certain advantages in classification accuracy, kappa value, and feature distribution, which is expected to provide ideas for the research and development of MI-BCI online systems based on EEG with fewer channels.

## Data Availability Statement

The original contributions presented in the study are included in the article/supplementary material, further inquiries can be directed to the corresponding authors.

## Ethics Statement

Ethical review and approval was not required for the study on human participants in accordance with the local legislation and institutional requirements. Written informed consent from the patients/participants or patients/participants’ legal guardian/next of kin was not required to participate in this study in accordance with the national legislation and the institutional requirements.

## Author Contributions

FW: conceptualization, methodology, writing—original draft, software, and validation. HL: methodology and conceptualization. LZ: investigation and data curation. LS: data curation and software. JZ: software. AG: writing—review and editing, and funding acquisition. YF: conceptualization, methodology, writing—review and editing, and funding acquisition. All authors contributed to the article and approved the submitted version.

## Conflict of Interest

The authors declare that the research was conducted in the absence of any commercial or financial relationships that could be construed as a potential conflict of interest.

## Publisher’s Note

All claims expressed in this article are solely those of the authors and do not necessarily represent those of their affiliated organizations, or those of the publisher, the editors and the reviewers. Any product that may be evaluated in this article, or claim that may be made by its manufacturer, is not guaranteed or endorsed by the publisher.
